# Novel Chronic Wound Healing by Anti-biofilm Peptides and Protease

**DOI:** 10.34172/apb.2022.047

**Published:** 2021-03-27

**Authors:** Fatemeh Sadat Ghoreishi, Rasoul Roghanian, Giti Emtiazi

**Affiliations:** Department of Cell and Molecular Biology & Microbiology, Faculty of Biological Science and Technology, University of Isfahan, Isfahan, Iran.

**Keywords:** Antimicrobial peptides, Bacteriocin, Biofilm, Metalloprotease, Wound healing

## Abstract

Chronic wounds have made a challenge in medical healthcare due to their biofilm infections, which reduce the penetrance of the antibacterial agents in the injury site. In infected wounds, the most common bacterial strains are *Staphylococcus aureus* and *Pseudomonas aeruginosa*. Biofilm disruption in chronic wounds is crucial in wound healing. Due to their broad-spectrum antibacterial properties and fewer side effects, anti-biofilm peptides, especially bacteriocins, are promising in the healing of chronic wounds by biofilm destruction. This study reviews the effects of antimicrobial and anti-biofilm agents, including bacteriocins and protease enzymes as a novel approach, on wound healing, along with analyzing the molecular docking between a bacterial protease and biofilm components. Among a large number of anti-biofilm bacteriocins identified up to now, seven types have been registered in the antimicrobial peptides (AMPs) database. Although it is believed that bacterial proteases are harmful in wound healing, it has recently been demonstrated that these proteases like the human serine protease, in combination with AMPs, can improve wound healing by biofilm destruction. In this work, docking results between metalloprotease from *Paenibacillus polymyxa* and proteins of *S. aureus* and *P. aeruginosa* involved in biofilm production, showed that this bacterial protease could efficiently interact with biofilm components. Infected wound healing is an important challenge in clinical trials due to biofilm production by bacterial pathogens. Therefore, simultaneous use of proteases or anti-biofilm peptides with antimicrobial agents could be a promising method for chronic wound healing.

## Introduction

Skin is the largest organ in our body that acts as a physical barrier and protects it from environmental threats. However, wounds and injuries can suppress the health and skin defense in the face of pathogens. The skin consists of two tissue layers, namely epidermal cells, saturated with keratin and a thin bottom layer from the connective tissue.^1^ In natural conditions, skin injuries are repaired completely by the wound healing process.^2^ Skin damage repair is an active process including three overlapping stages (i) Inflammation stage, with increasing the blood components in the wound site, resulting in platelet accumulation, blood coagulation, and inflammatory cell migration to the injury site. (ii) Reproduction stage, with the migration and proliferation of keratinocytes, fibroblasts, and epithelial cells, which cause tissue recovery and granulation. (iii) Remodeling stage, which causes tissue structural integrity and functional competence. In some cases, such as immunocompromised patients,^2^ however, these wounds are infected by bacterial pathogens having biofilms, resulting in a chronic infected wound.^3^ This biofilm inhibits the antibacterial agent penetration to the wound site.^4^ Chronic wounds are the result of gradual regeneration of tissue by biochemical agents, such as proteolytic enzymes, that making difficulties in the wound healing treatment. These wounds are recognized by some characteristics, such as flawed tissue, debris, bacterial biofilm production, long-term inflammation, and moisture imbalance. Therefore, biofilm removal in chronic wounds is critical to accelerating wound healing.^2^

According to these findings, a combination of antibiotics and anti-biofilm agents, to utilize their synergic effects, is the most effective strategy to wound healing management. As it was shown that chronic wound healing, when combined with anti-biofilm agents, could overcome incurable wounds, the use of antibiotics declined by 25% during the 4-year study period.^3^ A recent study has also shown that the use of anti-biofilm agents can facilitate improvements in the healing status of most hard-to-heal wounds.^5^

In the present study, the effect of the antimicrobial peptides (AMPs), especially anti-biofilm bacteriocins, and proteases as a novel method are reviewed in wound healing.

## Biofilms in chronically infected wounds

Biofilm is more resistant to antibiotics and the host immune system than the planktonic cells, which suggests altered metabolic activity in the biofilm cells. In a study, it was found that gene expression patterns in several gene groups were different in two of these growth conditions. Increasing gene expression levels of the attachment proteins, those involved in the biosynthesis of murein, glucose amino glycan polysaccharides, and other enzymes responsible for the envelope functions suggest the high activity of the envelope in the biofilm. Furthermore, evidence has shown that format fermentation, urease activity, oxidative stress responses, and, as a consequence, the acid and ammonium production increase in the biofilm cells.^6^

The importance of the biofilm has been enhanced in clinical treatment since it has been identified as one of the most important factors in wound healing delay. Biofilm protects bacterial biomass against antibiotics. With increasing obesity, diabetes epidemic, and population aging, chronic wounds such as diabetic foot ulcers, make a major problem in clinical trials. Recent studies have shown that chronic wound healing depends on the biofilm production within the wounds. Biofilm production in chronic wounds is initiated by bacterial attachment to the biotic/abiotic surfaces, resulting in slow improvement in wound healing, immune system disorders, and bacterial resistance to antibiotics. Biofilm has been detected in 60% of chronic wounds and is considered to be an agent of slowing down or inhibiting wound healing.^3^

## Common pathogenic bacterial strains in infected wounds

Recent studies have shown that there are various kinds of pathogenic bacteria associated with wound infections. The most common bacterial strains, isolated from infected wounds, are *Staphylococcus, Enterococcus, Enterobacter, Pseudomonas,* and *Finegoldia.* For example, diabetic foot ulcer is commonly infected by *Staphylococcus aureus*, which, together with *Pseudomonas aeruginosa*, are generally the most common strains isolated from infected wounds.^7^

### S. aureus and its biofilm

*S. aureus* is an opportunistic pathogen known as a major cause of wound and skin infection. This bacterium belongs to the natural microbiome of the nasopharynx, eye, skin, intestine, and urogenital tract.^8^

*S. aureus* is an important agent of community-associated (CA) infections, a gram-positive human pathogen that produces a wide range of toxins and enzymes enabling it to produce acute infections, such as bacteremia, skin abscess, and skin infection. Besides, most strains of this pathogen can produce biofilm and become persistent in human tissues, causing chronic infections. Biofilm-associated infections are hardly treated and generally need long-term antibiotic therapies. Bacterial biofilm resistance to the antibiotics is due to slow bacterial growth, cell phenotype heterogeneity, producing persistent cells, and inactivation or reduction of antibiotic penetrance. The slow antibiotic penetration into the biofilm suggests that the cells may be facing the subinhibitory concentrations of antibiotics. Biofilm production is done by at least three stages, such as initial attachment, biofilm maturation, and biofilm distribution. Initial surface attachment is dependent on the bacterial surface molecules, such as *S. aureus* murein hydrolase (AltA), teichoic acid, and Fibronectin-Binding Proteins (FnBPs). The cells then proliferate and produce exopolysaccharides (EPS). The biofilm matrix of most clinical and laboratory staphylococcal strains consists of a large amount of the extracellular DNA (eDNA), which is attacked by bacterial nuclease Nuc1. The negative charge of this eDNA plays an important role in biofilm attachment and maturation. The eDNA release into the biofilm matrix depends on the murein hydrolase. AltA is the major autolytic enzyme in *S. aureus*, which is necessary for cell wall division and bacterial lyses. Moreover, biofilm autolysis in this strain is done and regulated by the Cid/Lrg holing-antiholin activity system. CidA is oligomerized in the bacterial cell membrane and increases the murein hydrolase activity by releasing out of the cells. In the disturbation stage, bacterial cells are separated from the biofilm and spread in the new sites for producing a new infection. A quorum-sensing system (Agr) is responsible for switching between biofilm production and bacterial spread. Quorum sensing controlled Phenol-soluble modulins (PSMs) are present as amyloid fibers in the biofilm that have dual activities in the biofilm sustainability in certain conditions and biofilm spread due to their surfactant-like activity.^4^ Other proteins involved in the biofilm production in *S. aureus* include the clumping factor B (*clfB*), Ser-Asp-rich fibrinogen-binding bone sialoprotein-binding protein (*sdrC*),^6^ and surface protein G (*sasG*).^9^

The biofilm phenotype variation in methicillin-resistant *S. aureus* (MRSA) and methicillin-susceptible *S. aureus* (MSSA) depends on the IcaABCD operon that produces PIA. Unlike MSSA, the biofilm production in MRSA strains is Ica-independent and is related to the attached surface proteins, such as FnBPs, and eDNA.^10^

### P. aeruginosa and its biofilm

*P. aeruginosa* is a widespread bacterium present in various environments, such as soil, freshwater, and marines. It is also an opportunistic pathogen in the airways of patients with cystic fibrosis (CF) and immune suppression diseases, such as acquired immunodeficiency syndrome (AIDS), cancer, and burns. Its virulence factors are controlled by the quorum-sensing pathway (LasI). This bacterium has a global regulatory system, the quorum sensing, which controls the gene expression of some virulence factors.^11^

The cells of *P. aeruginosa* are trapped by a gelatinous polymeric matrix of alginate. The biofilm in this bacterium is resistant to antibiotics, which causes chronic infections in the urinary tract and epithelium of the lungs in CF patients. Due to the lack of *in situ* studies about the production of this matrix, the inhibition of biofilm is a major problem.^12^

Biofilm production in *P. aeruginosa* includes the following steps: cell attachment and proliferation, microcolony production, and finally evolution and differentiation to the mature biofilm, with a specific structure and resistance to antibiotics.^13^

In the initial stages of the biofilm production, the cells quickly attach to the surface by improving the *algC* gene expression. Cells with lower expression of algC have been shown to have less ability to remain connected to the surfaces.^12^

In this bacterium, several virulence factors are controlled by the quorum-sensing pathway. The LasI expression progressively decreased along with the biofilm production, while the expression of the rhlI gene remained stable, but it was expressed in fewer cells. Spatial studies showed that these two genes were maximally expressed in the bottom layers of the biofilm, and their expression decreased by increasing the biofilm thickness.^14^ Furthermore, the *pel* gene family is involved in the early and late stages of biofilm production.^15^ Other proteins involved in biofilm formation in this strain are PslG,^16^ and lectin.^17^

## AMPs

AMPs, low molecular weight peptides, are an integral part of the innate immune system, which are present in multicellular organisms (such as insects, vertebrates, plants, and humans) and such microorganisms as bacteria. AMPs have a wide range of antimicrobial activities, such as bacterial killing, and their activity is not inhibited by biological fluids and biofilms. They act in various sites within the cells, which reduce bacterial resistance to them.^2^

AMPs act in various sites within the microbial cells in a short time (even shorter than a bacterial cell division cycle). Other benefits are low bacterial resistance to them, widespread activity against resistant pathogens, both bactericidal and bacteriostatic properties, and the lack of inhibition of their activity by the fluids, secretions, and biofilms. More importantly, AMPs are present in all stages of wound healing, which demonstrates their simultaneous roles in this process.^2^

### Bacteriocins, bacterial AMPs

Bacteriocins are low-molecular-weight AMPs, which kill closely related bacteria.^18^ These peptides are produced by Gram-positive bacteria, such as *Lactobacillus, Lactococcus, Streptococcus, Enterococcus, Leuconostoc, Pediococcus,* and *Propionibacterium*, as well as by gram-negative strains, including *Escherichia coli, Shigella, Serratia, Klebsiella,* and *Pseudomonas.* Due to the structural and functional similarities, these AMPs belong to antibacterial components, including defencines, tionines, mangaines, and melittin, which are produced respectively by mammals, plants, frog, and bee venom. A few hundreds of bacteriocins have been characterized up to now.^19^ Iran is an ecosystem rich in various types of bacteria with different properties, among which bacteriocin-producing bacteria are very important. Examples of these include a bacteriocin with broad antimicrobial activity obtained from newly isolated nitrogen-fixing *Bacillus* strains in Iran.^20^ Besides, thermostable bacteriocins from *Bacillus pumilus* ZED17 and DFAR8 strains with antifungal activity were characterized in another study.^21^ A new hallucin isolated from *Halarchaeum acidiphilum* ASDL78 with antibacterial properties was identified recently.^22^

### AMPs in wound healing

To date, 23 AMPs with wound healing activity have been recognized and registered in the AMPs database, and nisin A is the only one with a bacterial origin.

#### Human Endogenous AMPs in wound healing

In every stage of the wound healing process, the wound site is attacked by molecules, which are responsible for inducing the wound healing process. One of these molecules is AMPs, which belong to the innate immune system. After skin injury, activated proteases release heparin-binding epidermal growth factors (HB-EGF) and amphiregulin, which have antimicrobial activity and are responsible for stimulating the expression of epidermal AMPs in the following stages. Complement and coagulation cascades are activated along with homeostasis, resulting in the cleavage of several proteins, such as fibrinogen or thrombin. The breakdown components of these proteins lead to increases in several AMPs, such as C3a, which is characterized by its antimicrobial activity.^2^

In the inflammation stage, the wound site is attached initially by neutrophils and then by monocytes and lymphocytes. Neutrophils are the most important producers of AMPs in the inflammation stage, which contain defensins (human neutrophil peptides), cathelicidins, and calgranulins in their cytosol. Cathelicidin is produced by proteinase3, after releasing of the granules, and is changed to the LL37 AMP. Cathelicidin is also responsible for the monocyte recruitment to the wound site to induce the vascular endothelial growth factor expression, resulting in keratinocyte migration to the wound site.^2^

In the proliferation stage, most AMPs are derived from epidermal keratinocytes. LL37 is maximally expressed in this stage. Due to the similar ancestral genes of defensins, neutrophils (in the inflammation stage) and keratinocytes (in the proliferation stage) produce and release similar AMPs, though the expression of AMPs is stage-dependent.^2^

Tissue growth and remodeling are associated with AMP expression. Epidermal AMPs, involved in wound healing, have a wide range of antibacterial activity: nBD-3 and RNase7 act extensively against *S. aureus*, psoriasin is effective against *E. coli*, and calgranulin can act against *Candida albicans*.^2^

Tissue remodeling is the latest stage of wound healing. Although there is currently no evidence for AMP production along this stage, the expression of collagen type VI, with high antimicrobial activity, has been shown to increase in this phase, which protects the skin and the connective tissue.^2^

## Anti-biofilm peptides

Some AMPs have anti-biofilm property, hence they are called anti-biofilm peptides. These are very promising for the treatment of wound infections containing biofilms and can interfere with biofilms in various stages.^23^ Totally, 59 peptides with anti-biofilm activity have been sequenced and registered in the AMPs database, seven of which have dual anti-biofilm and wound healing activities (Table 1).

**Table 1:** AMPs, with dual anti-biofilm and wound healing activities, registered in the AMPs database

**APD ID**	**Name**	**Source**
AP00150	Indolicidin	bovine neutrophils, *Bos taurus*
AP00205	Nisin A	*Streptococcus lactis* reclassified as *L.lactis*
AP00283	Human beta defensing 3	skin, tonsils, oral/saliva, *Homo sapiens*
AP00310	LL-37	Mesenchymal Stem Cells; islets; skin, sweat; airway surface liquid, saliva; *H. sapiens*; Also *Pan troglodytes*
AP01578	Myxinidin	Epidermal mucus, *Myxine glutinosa*
AP01976	Coprisin	Dung Beetle, *Copristripartitus*
AP02872	Esculentin 1-21	artificial, template derived

### Anti-biofilm synthetic peptides

DRGN-1 synthetic peptide, derived from the natural vk25 peptide, has multiple properties. This artificial AMP has antimicrobial and anti-biofilm activities against *P. aeruginosa* and *S. aureus* through permeabilized bacterial membranes and improves wound healing through stimulating the keratinocyte migration to the wound site and activating the wound healing pathways.^24^

Another synthetic peptide, PR557, is derived from human endogenous AMPs cathelicidin LL37, and tachyplesin1, which can destroy the biofilm and pre-biofilm of the MRSA.^25^

### Anti-biofilm bacteriocins

EPL or ε-poly-L-lysin is a natural AMP is produced by *Streptomyces albus.* This AMP has an antimicrobial effect against gram-positive and Gram-negative bacteria, as well as against *K.albicans.* This peptide is destructive, non-toxic, and inexpensive, which could be administrated in the form of oral consumption. In a novel study, a hydrogel was produced for the first time by the EPL attached to the catechol, which has anti-MRSA and anti-methicillin resistant *Acinetobacter baumannii* activity. This combination has been shown to have antibacterial and anti-biofilm activities.^26^

In a study in 2015, a novel bacteriocin was obtained from *Lactobacillus gasseri* SF, which showed significant effects against *Enterococcus faecalis*. This bacteriocin is heat-stable with a 3.5 kD molecular weight, which belongs to the class II bacteriocins, named bacteriocin SF. Bacteriocin SF down-regulates the mRNA expression of the *fsr* in the quorum-sensing pathway and biofilm-associated genes in *E. faecalis.* It had *in vitro* wound healing properties via increasing the proliferation and migration of the HaCaT cells, enhancing the expression of the fibroblast growth factor receptor 2-IIIb (FGFR2-IIIb), transforming growth factor-beta (TGF-β1), interleukin-8 in the HaCaT cells, and anti-biofilm activity.^27^

So far, a large number of anti-biofilm bacteriocins have been identified against *P. aeruginosa* and *S. aureus* (Table 2),^28-42^ and other species (Table 3).^30,42-64^

**Table 2:** Anti-biofilm bacteriocins against *S. aureus* and *P. aeruginosa*

**Bacteriocin**	**Producer strain**	**Biofilm target**	**Reference**
Sonorensin	*Bacillussonorensis* MT93	*S. aureus*	^27^
Colicin-like bacteriocin	*P. aeruginosa* and *E. coli*	Sensitive species of *P. aeruginosa* and other genera	^28^
Licheniocin 50.2	*Bacillus licheniformis* VPS50.2	*S. aureus*	^29^
Bacteriocin HW01	*Pediococcusacidilactici* HW01	*P. aeruginosa*	^30^
Bacteriocin	*Leuconostocmesenteroides* CHBY46	*P. aeruginosa* and *S. aureus*	^31^
Gallidermin	*Streptococcus gallinarum*	MRSA	^32^
Hyicin 4244	*S. hyicus* 4244	*S. aureus*	^32,33^
Lysostaphin	*Streptococcus simulans* biovar *staphylolyticus* ATCC1362	*S. aureus*	^32^
Bacteriocin	*Lactobacillus acidophilus* (*L. acidophilus*)	*P. aeruginosa*	^34^
Bacteriocin	*Enterococcusitalicus* ONU547	*P. aeruginosa* & other genera	^35^
Bacteriocin	*P. aeruginosa*	MRSA	^36^
Bac F1	*Lactobacillus plantarum* subsp. *argentoratensis* SJ33	*S. aureus* and *P. aeruginosa*	^37^
Bac F2	*L. plantarum* subsp. *argentoratensis* SJ33	*S. aureus* and *P. aeruginosa*	^37^
Plantaricin GZ1-27	*L. plantarum*	MRSA	^38,39^
BaCf3	*Bacillus amyloliquefaciens* BTSS3	*P. aeruginosa*	^40^
BL8	*B. licheniformis* BTHT8	*P. aeruginosa* & other genus	^41^

**Table 3:** Anti-biofilm bacteriocins against other species

**Bacteriocin**	**Producer strain**	**Biofilm target**	**Reference**
BGBU1-4	*L.lactis*	*Listeria monocytogenes*	^29^
Curvatus LHM	*Lactobacillus curvatus*	*Streptococcusmutans* and *Streptococcus sanguinis*	^42^
Bacteriocin	*Proteus mirabilis*	*Klebsiella, Proteus,* and *E. coli*	^43^
EntV	*E.faecalis*	*K.albicans*	^44^
CFS^*^	*L.lactis* ALB79	*L. monocytogenes*	^45^
Nisin A	*L. lactis* sp. UQ2	*L. monocytogenes*	^45^
CFS^*^	*Lactobacillus eurvatus* ET06, ET31	*L. monocytogenes*	^45^
CFS^*^	*Lactobacillus fermentum* ET35	*L. monocytogenes*	^45^
CFS^*^	*Lactobacillus delbrueckii* ET32	*L. monocytogenes*	^45^
CFS^*^	*L. curvatus* ET31	*L. monocytogenes*	^45^
CFS^*^	*L. plantarum* ST8SH	*L. monocytogenes*	^45^
Bacteriocin	*Lactobacillus sakei*	*L. monocytogenes*	^45^
CFS^*^	*P.acidilactici* ET34	*L. monocytogenes*	^45^
Bacteriocin	*Enterococcus faecium*	*L. monocytogenes*	^45^
CFS^*^	*E. faecium* ET05, ET12 and ET88	*L. monocytogenes*	^45^
Enterocin AS-48	*E. faecalis*	*L. monocytogenes*	^45^
Bacteriocin	*Citrobacter freundii*	*E. coli, Citrobacter* sp., *K. pneumonia*	^46^
CFS^*^ 22 CFS^*^ 24	*E. faecium*	*Listeria* sp.	^47^
CFS^*^ 27	*E. faecalis*	*Listeria* sp.	^47^
GAM217	*L. lactis* strain GAM217	*E. coli* and *Staphylococcus epidermidis*	^48^
Lactocin AL705	*Lactobacillus casei* CRL705	*L. monocytogenes*	^49,50^
Bacteriocin	*Bacillus subtilis* GAS101	*S. epidermidis* and *E. coli*	^51^
BM1157	*Lactobacillus crustorum*	*L. monocytogenes*	^52^
DF01	*Lactobacillus brevis*	*E. coli* and *Salmonella typhimurium*	^53^
Bacteriocin	*L. acidophilus* ATCC4356	*B. subtilis*	^54^
BMP11	*L. crustorum* MNO47	*Citrobacter sakazakii*	^55^
Bacteriocin	*L. sakei* CRL186	*L. monocytogenes*	^56^
Bacteriocin	Marine *Bacillus* sp. strain Sh10	*P.mirabilis*	^57^
Nisin Z	*L. lactis* B313	*L. monocytogenes*	^58^
Lichenicidin	*B. licheniformis*	*L. monocytogenes*	^58^
Subtilomycin	*B. subtilis* T2-shier-5	*L. monocytogenes*	^58^
Subtilomycin	*B. subtilis* VKK-2NL	*L. monocytogenes*	^58^
Lichenicidin	*B. licheniformis* HBH3-1	*L. monocytogenes*	^58^
Lichenicidin	*B. sonorensis* VITM31	*L. monocytogenes*	^58^
Subtilomycin	*B. subtilis* B.Pat.23	*L. monocytogenes*	^58^
Bacteriocin	*L. acidophilus* ATCC4356	*Serratia marcescens*	^59^
Bacteriocin	*L. plantarum* ATCC8014	*S.marcescens*	^59^
Bac LP17	*Enterococcusmundtii* LP17	*L. monocytogenes*	^60,61^
WH01	*P.acidilactici*	*E. faecalis*	^62^
BL8	*B. licheniformis* BTHT8	*Bacillus altitudinis,* *B. pumilus, Brevibacteriumcasei, Streptococcus warneri, Micrococcus luteus, Bacillus niacicini, Geobacillus stearothermophilus*	^41^
Bacteriocin	*L. plantarum* ST8SH	*L. monocytogenes*	^63^

^*^CFS: cell free supernatant.

So far, seven anti-biofilm bacteriocins have been sequenced and registered at the AMPs database, including nisin A, colistin A, gramicidin S, enterocin O16, hyicin, polymyxin B, and VLL-28 (Table 4), all of which have been explained in the following subtitles. Nisin A is the only wound healing AMP, derived from bacteria (bacteriocin), which has dual wound healing and anti-biofilm activities. Thus, using these anti-biofilm bacteriocins could be effective indirectly in chronic wound healing, which contains bacterial biofilms.

**Table 4:** Bacteriocins registered on the AMPs database, with anti-biofilm activity*

**APD ID**	**Name**	**Source**	**Sequence**	**Net charge**	**Hydrophobic residue%**	**Activity**
AP00205	Nisin A	*S.lactis*, reclassified as *L.lactis*	ITSISLCTPGCKTGALMGCNMKTATCHCSIHVSK	3	44%	Anti-Gram+, Spermicidal, Antibiofilm, Wound healing, Anticancer
AP02204	Colistin A	*P.polymyxa* var. colistinus; Also known as *B.polymyxa*	KTKKKLLKKT	6	20%	Anti-Gram-, anti-sepsis, Antibiofilm
AP02243	Gramicidin S	*Aneurinibacillusmigulanus*(former *B.brevis*)	VKLFPVKLFP	2	60%	Anti-Gram+ & Gram-, Antifungal, Spermicidal, Hemolytic, Antibiofilm
AP02520	Enterocin O16	*E.faecalis*	LGSCVANKIKDEFFAMISISAIVKAAQKKAWKELA VTVLRFAKANGLKTNAIIVAGQLALWAVQCGLS	6	58%	Anti-Gram+, Antifungal, Antibiofilm
AP02925	Hyicin 4244	*S. hyicus* 4244	NKGCSACAIGAACLADGPIPDFEVAGITGTFGIAS	-2	51%	Anti-Gram+, Antibiofilm
AP02928	Polymyxin B	*B.aerosporus* Greer	KTKKKFLKKT	6	20%	Anti-Gram-, Antifungal, anti-sepsis, Antibiofilm
AP03049	VLL-28	*S. islandicus*	VLLVTLTRLHQRGVIYRKWRHFSGRKYR	10	35%	Anti-Gram+ & Gram-, Antifungal, Antibiofilm, Anticancer

*The bolded APD ID is regarding the bacteriocin with dual anti-biofilm and wound healing activities.

#### Nisin

Nisin is a lantibiotic that belongs to the bacteriocins and is characterized by its antibacterial properties against Gram-positive bacteria and the lack of antibacterial activity against Gram-negative bacteria. One of the most important features of nisin is the 34-amino acid sequence, which is dehydrated in serine and threonine residues. This post-translational modification produces unusual amino acids, dehydroalanine, and dehydrobutirine before nisin maturation.^65^

This bacteriocin is produced by *Lactococcus lactis* and has unusual amino acids, such as lanthionine and methyllanthionine, which are crucial for its bacterial producer. This peptide has been approved, by the Food and Drug Administration (FDA) and World Health Organization (WHO) in 1953 and has been used as a food additive.^66^

In a recent study, it has been shown that nisin A significantly affects the migration of the human umbilical vein endothelial cell and the HaCaT cells. This study has suggested that nisin A can be a potential treatment for wound healing, as it increases the motility of the skin cells and decreases bacterial growth in the infected wound.^65^

**Figure 1:**
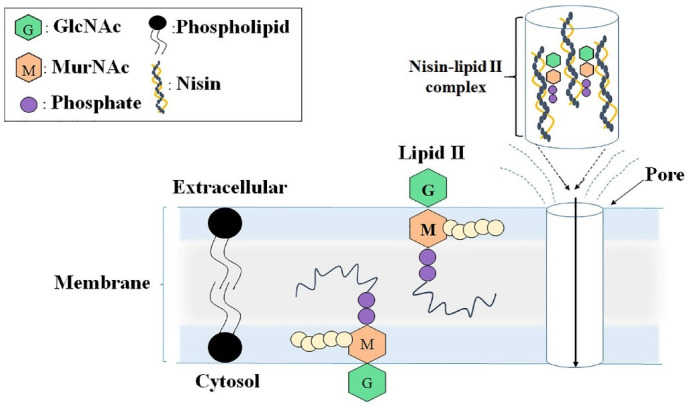
Schematic diagram of the antibacterial mechanism of nisin, including the pore formation in the cell surface, by creating a complex between nisin and bacterial membrane lipid II. GlcNAc: N-Acetylglucosamine; MurNAc: N-Acetylmuramic acid.

Nisin is the most well-known bacterocin. This lantibiotic forms complexes with bacterial lipid II (in gram-positive bacteria), resulting in the inhibition of the cell wall biosynthesis and increasing the cell membrane permeability (Figure 1).^67^

#### Colistin

Discovered in the 1940s, colistin is a polymyxin antibiotic against Gram-negative infections, which is produced by *Bacillus polymyxa*. This peptide is a detergent-like molecule that has a bactericidal effect and its application was the last resort for the treatment of multidrug-resistant infections, including *P. aeruginosa*, *A. baumannii*, and *Klebsiella pneumonia.* Colistinformulations in clinical usage are colistin sulfate and colistin methane sulfonate.^68^

The mechanism of its antibacterial action against Gram-negative bacteria is through its detergent-like effect via two steps. The initial binding is done with electrostatic interaction between the polycationic ring of colistin to the anionic components of the cell envelope, leading to the disruption of the outer membrane, thus killing the bacterium.^69^

#### Gramicidin S

Gramicidin S is a peptide produced by *Aneurinibacillusmigulanus* (formerly *B. brevis*), which displays strong hemolytic potential. It delocalizes peripheral proteins involved in the cell division and cell wall synthesis, but has no effect on integral membrane proteins or DNA.^70^

#### Polymyxin B

Polymyxin B is a peptide that is used clinically. Polymyxins interact with lipopolysaccharide (LPS) in the outer membrane of Gram-negative bacteria. The polycationic ring of polymyxins binds to the outer membrane, which displaces the calcium and magnesium bridges responsible for stabilizing the LPS. Therefore, polymyxins make a disruptive effect, leading to permeability changes in the outer membrane and cell death.^71^

#### Hyicin 4244

Hyicin, a small AMP with widespread antibacterial activity, has been found in the supernatant culture of the *Streptococcus hyicus* 4244. The organization of its gene cluster (hyiSABCDEFG) is similar to that of subtilosin A, and at least 25% of its proteins are similar to those encoded by subtilosin A gene cluster. A study has shown that this bacteriocin acts against 14 Staphylococcal strains, isolated from human infections and bovine mastitis, all of which are biofilm producers. Furthermore, this AMP inhibits planktonic cells, pre-biofilms, and mature biofilms in these strains.^72^

#### VLL-28

In a study in 2015, a novel cyclic-AMP (cAMP)-like peptide, VLL-28, was isolated and characterized in *Sulfolobusislandicus*, which had widespread antibacterial activity. The mechanism of this peptide is to be localized not only on the cell membrane but also in the cytoplasm; it can also bind to nucleic acids. Thus, this peptide can apply its antibacterial activity by triggering the membrane and intracellular targets. VLL-28 is the first example of the archaeal AMP (archaeocin) against *Candida* spp. biofilms.^73^

#### Enterocin O16

In a study in 2015, a novel bacteriocin, enterocin O16, was isolated from *E. faecalis,* with a molecular weight of 7231 Da and a 29 amino acid sequence corresponding to the residues 90-119 in EF_1097 protein. The *gelE* gene in the *fsr* quorum-sensing system was demonstrated to be essential for producing and regulating this AMP.^74^

**Figure 2:**
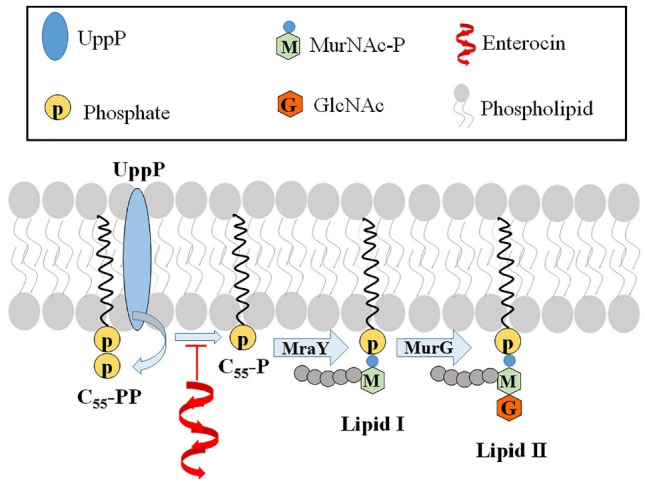
Schematic diagram of the mechanism of the enterocin antibacterial action: inhibition of UppP enzyme involved in phosphate supply for bacterial wall synthesis. UppP: Undecaprenyl diphophate phosphatase; GlcNAc: N-Acetylglucosamine; MurNAc-P: N-Acetylmuramic acid-phosphate; MraY: translocase enzyme; MurG: an enzyme catalyses the transfer of GlcNAc residue from lipid I to lipid II.

The anti-bacterial mechanism of the enterocin 1070 is receptor-mediated binding. This bacteriocin inhibits the undecaprenyl diphosphate phosphatase (UppP), an enzyme involved in cell wall synthesis (Figure 2).^75^

### Anti-biofilm peptides from extremophiles

The extreme environments have become an important source for the identification of novel bacterial metabolites and peptides with antimicrobial activity. These antimicrobial metabolites have not been explored as widely as those of the mesophilic microorganisms. In a research, cyclic lipopeptides isolated from polar marine bacteria genera, *Pseudoalteromonas* and *Psychromonas*, did not possess antimicrobial activity, but some of them had strong anti-biofilm activity against *S. aureus*.^76^ A cyclic dipeptide was also isolated from *Halobacillussalinus* with anti-quorum quenching and anti-biofilm properties.^77^

## Novel approaches in the healing of chronic wounds containing biofilm

**Figure 3:**
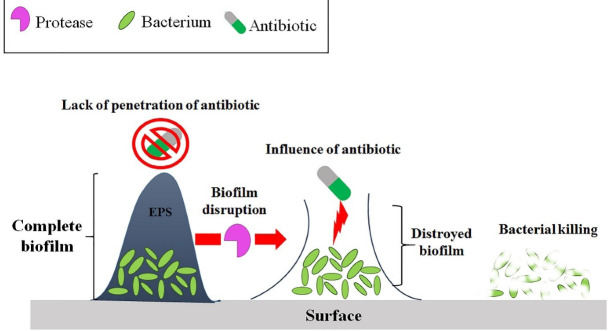
Schematic of how protease works against biofilm. In the presence of biofilm, antibiotics can not to penetrate and kill the cells. After the biofilm is destroyed by the protease enzyme, the penetrating antibiotic kills the bacteria. EPS: Exopolysaccharides.

Biofilm is surrounded by EPS, which consists of biopolymers, such as EPS, nucleic acids, lipids, and proteins. Hence, biofilm destruction is a good idea for facilitating chronic wound healing by antimicrobial agents. Protease enzymes are one of the good candidates for biofilm destruction of pathogenic bacteria (Figure 3). So far, many studies have been performed on the effect of protease on biofilms. For example, a metalloprotease obtained from *Halobacilluskarajensis* has been confirmed to have an anti-biofilm effect.^78^ Besides, protease enzymes were immobilized on chitosan for the development of anti-biofilm properties.^79^ Furthermore, the effects of proteases as anti-biofilm agents have been confirmed in the food industry.^80^ Extracellular proteases of *Actinomycetes* inhibits *S. aureus* biofilm formation.^81^ similarly, the protease enzyme of a skin commensal fungus attenuates *S. aureus* biofilm formation.^82^ Few studies, however, have been done on the positive effect of proteases on wound healing. In a recent study in 2019, for example, a hydrogel containing antibacterial agent has been stabilized on the protease (from *B. licheniformis*) as a carrier made by nanotechnology. This combination of antibacterial and anti-biofilm agents treated infected chronic wounds. The application of this conjugation with ciprofloxacin enhanced the bactericidal effect of this antibiotic.^83^

As mentioned above, using synergetic effects of the antibacterial agents and proteases could be a promising method for chronically infected wound healing. There are limited and novel studies about the effect of proteases on wound healing. Thus, docking analysis could be useful for understanding the interactions between proteases and bacterial components involved in the biofilm structure. Therefore, the crystal structures of Gentlyase, the neutral metalloprotease of *P.polymyxa*, the enterocin O16, biofilm-associated agents in *S. aureus*, including the clumping factor B (ClfB), Ser-Asp-rich fibrinogen-binding bone sialoprotein-binding protein (SdrC),^6^ PSMs,^4^ surface protein G (SasG),^9^ and biofilm-related components in *P. aeruginosa*, such as alginate (AlgC),^12^ glycoside hydrolase (PslG),^16^ sodium alginate,^84^ and lectin,^17^ were obtained from RCSB and Pubchem databases. Docking analysis was done by the Molegro Virtual Docker 2013 v6.0.1, with MolDock SE algorithm and energy threshold 100. The effects of the metalloprotease of *P.polymyxa* and the enterocin O16 were analyzed on the biofilm-associated agents.

According to the docking results (Tables 5 and 6), the metalloprotease interacts effectively with ligands involved in the biofilm formation in *S. aureus* and *P. aeruginosa*. There is also an effective interaction with enterocin, as an anti-biofilm bacteriocin. Furthermore, although enterocin has no effects on gram-negative bacteria, it was effective against gram-negative bacterial biofilms.

**Table 5:** The results of docking analysis between metalloprotease of *P. polymyxa* (as a protein) and ligands involved in biofilm formation in *S. aureus* and *P. aeruginosa*

**Strain**	**Ligand**	**Binding energy (kcal/mol)**	**Possible amino acids involved in the interaction**
*S. aureus*	PSM	-181	Gly, His, Glu, Tyr, Asp, Asn, Val,
*P. aeruginosa*	Sodium alginate	-123	Gln, Gly, Glu,
	Lectin	-189	Gly, Asn, Tyr, Trp, Leu

**Table 6:** The results of docking analysis between enterocin (as a ligand) and proteins involved in biofilm formation in *S. aureus* and *P, aeruginosa*

**Strain**	**Protein**	**Binding energy (kcal/mol)**	**Possible amino acids involved in the interaction**
*S. aureus*	ClfB	-124	Thr, Asn, Gly, Asp, Ile, Lys, Arg, Ala
	SdrC	-171	Ile, Lys, Thr, Glu, Gln, Tyr, Asn, Val
	SasG	-100	Asp, Ile
	FnBP	-161	Gly, Arg, Ala, Lys, Asp
*P. aeruginosa*	AlgC	-165	Gly, Glu, Thr, Lys, Met, Phe
	PslG	-140	Glu, Gln, Leu, Gly, Ser, Arg,
	LasI	-138	Thr, Arg, Val, Phe
	PelB	-127	Trp, Phe, Gly,

The modeled structure of interactions with the highest energy interaction is designed by Molegro Virtual Docker (Figure 4). Also, the effective residues in these interactions are designed by Molegro software and shown in Figure 5.

**Figure 4:**
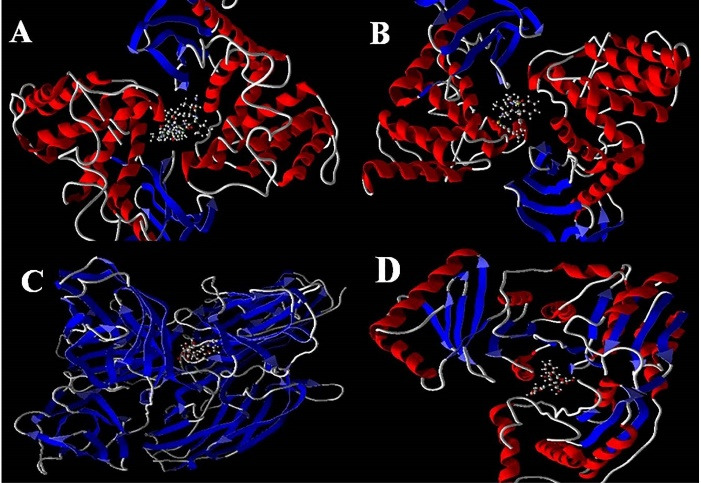
Modeled structure of the interaction between (A) metalloprotease from *P. polymyxa* and PSM protein in *S. aureus*; (B) metalloprotease from *P. polymyxa* and lectin in *P. aeruginosa*; (C) enterocin and SdrC in *S. aureus*; (D) enterocin and AlgC in *P. aeruginosa*, designed by Molegro Virtual Docker.

**Figure 5:**
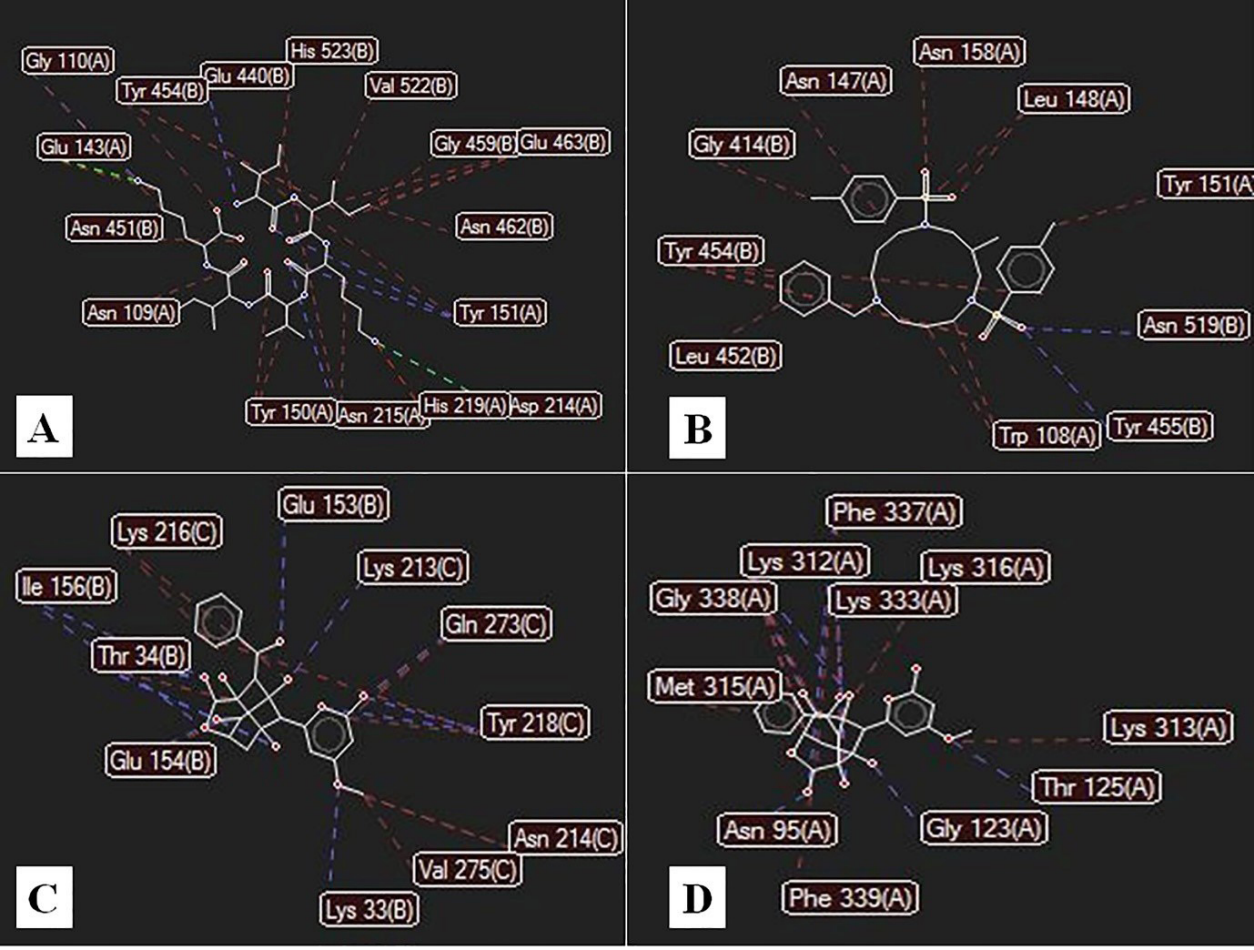
Two-dimensional diagram of the interactions with different residues in docking results between (A) metalloprotease from *P. polymyxa* and PSM protein in *S. aureus*; (B) metalloprotease from *P. polymyxa* and lectin in *P. aeruginosa*; (C) enterocin and SdrC in *S. aureus*; (D) enterocin and AlgC in *P. aeruginosa*, designed by Molegro Virtual Docker.

## Conclusion

This review discusses anti-biofilm bacteriocins and AMPs effective in wound healing, as well as the effect of protease enzymes on chronic wound healing as a novel method. Traditional and chemical antibacterial agents have failed in the face of infections due to resistance mechanisms in pathogens, such as biofilm production. Despite a large number of new antibacterial agents produced and identified every year, pathogens enhance their antibacterial mechanisms and stabilize infections. Consequently, it is promising to use the newer methods and synergetic effects of antibacterial and antivirulence agents. In fact, the use of antibacterial agents affecting the growth of the pathogen is not necessary for the fight against infections, and the use of antivirulence agents, such as anti-biofilm peptides, can also be effective. One of the most important virulence factors is biofilm production, which reduces the entry of antibacterial agents into the site of infection. Thus, the biofilm disruption could be very effective in the face of bacterial infection, especially wound healing. In fact, one of the most important reasons for the failure of wound healing is the presence of biofilm at the wound site. AMPs, low-molecular-weight peptides, have a wide range of antimicrobial activities, such as bacterial killing, and their activity is not inhibited by biological fluids and biofilms. They act in various sites within the cells, which reduces bacterial resistance to them. Bacteriocins, bacterial AMPs, are one of the most important and well-known AMPs that can be used for therapeutic purposes. So far, countless AMPs and bacteriocins have been identified with anti-biofilm properties, each of which can be investigated in wound healing due to the biofilm destruction. Among the seven anti-biofilm bacteriocins registered on the AMPs database, nisin is the only one with a dual anti-biofilm and wound-healing effect and is therefore more important. Besides, protease enzymes were previously believed to only have detrimental effects on wound healing, while today they have been shown to be able to accelerate the wound healing process by destroying the biofilm formed in chronic wounds. In conclusion, the combined use of anti-biofilm agents with antibacterial agents can accelerate wound healing and help treat chronic wounds.

Another suggested use of anti-biofilm peptides, bacteriocins, and protease enzymes may be in adjuvant therapy of patients with COVID-19. It has been shown that the formation of biofilms in the lungs and gastrointestinal tract has led to ineffective drugs in these patients.^85^ Therefore, the use of these anti-biofilm agents is recommended in the treatment of people with COVID-19.

## Acknowledgments

The authors of this article want to show their appreciation to the people of the Department of Microbiology, University of Isfahan, Iran.

## Ethical Issues

Not applicable.

## Conflicts of Interest

There are no conflicts of interest in this work.
